# A rare association of invasive infective endocarditis due to *Abiotrophia defectiva* with ventricular septal defect and recurrent Henoch-Schonlein purpura in a child

**DOI:** 10.1186/s13019-022-02092-2

**Published:** 2022-12-17

**Authors:** Wenpeng Dong, Shuibi Wu, Jing Zhou

**Affiliations:** 1 Department of Cardiovascular Surgery, The First Affiliated Hospital of Anhui Medical University, Jixi Road 218, Shushan District, Hefei, 230032 China; 2 Department of Cardiovascular Surgery, The Third People’s Hospital of Chengdu, Chengdu, China

**Keywords:** Infective endocarditis, *Abiotrophia defectiva*, Henoch-Schonlein purpura, Tricuspid valve replacement

## Abstract

**Background:**

Henoch-Schonlein purpura is the most common vasculitis in childhood, usually triggered by an upper respiratory tract infection and rarely observed in infective endocarditis patients. *Abiotrophia defectiva* is a rare causative agent of infective endocarditis associated with pre-existing heart disease, immunocompromised and prosthetic valves. Dental procedures are also a common predisposing factor.

**Case presentation:**

We present the first pediatric congenital heart disease case of infective endocarditis caused by *Abiotrophia defectiva* combined with recurrent Henoch-Schonlein purpura. A 10-year-old girl with uncorrected congenital heart defects and Henoch-Schonlein purpura developed a purple petechial rash again. Transthoracic echocardiography evaluation revealed multiple irregular vegetations on the right ventricular side of the ventricular septal defect and on the tricuspid valve leaflets. Blood cultures grew *Abiotrophia defectiva*. The girl received cardiac surgery for vegetation resection as well as congenital heart defect correction and tricuspid valve replacement. Five months after the surgery, the patient was in satisfactory condition without any signs of endocarditis or valve insufficiency and her purpuric rash disappeared.

**Conclusions:**

The coexistence of recurrent Henoch-Schonlein purpura and infective endocarditis is possible. *Abiotrophia defectiva* belongs to the streptococcus with a high virulence. In addition, cardiovascular surgery is often required for pediatric infective endocarditis associated with *Abiotrophia defectiva*, and bioprosthetic valve replacement is considered feasible for irreparable tricuspid valve in children.

## Introduction

Henoch-Schonlein purpura (HSP) is a common systemic vasculitis in childhood, characterized by palpable purpura on the lower limbs, arthralgia, abdominal pain and renal disease. Upper respiratory infections caused by streptococci and staphylococci are considered to be potential predisposing factors, while a few cases lack a clear trigger [1]. Infective endocarditis (IE) in combination with HSP is rare. IE is a fatal cardiac valve disease with an overall mortality rate of 20% at 30 days [2]. The most common organisms associated with IE are staphylococcus, streptococcus, and enterococcus species, while *Abiotrophia defectiva* rarely causes IE. In this paper, we report an extremely rare case of IE caused by *Abiotrophia defectiva* with ventricular septal defect (VSD) and recurrent HSP in a child and no cases involved the association of IE due to *Abiotrophia defectiva* with recurrent HSP in medical literature in English. Because of the large vegetations, VSD and irreparable tricuspid valve, vegetation eradication, congenital heart defect correction and tricuspid valve replacement were performed.

## Case report

**Fig. 1:**
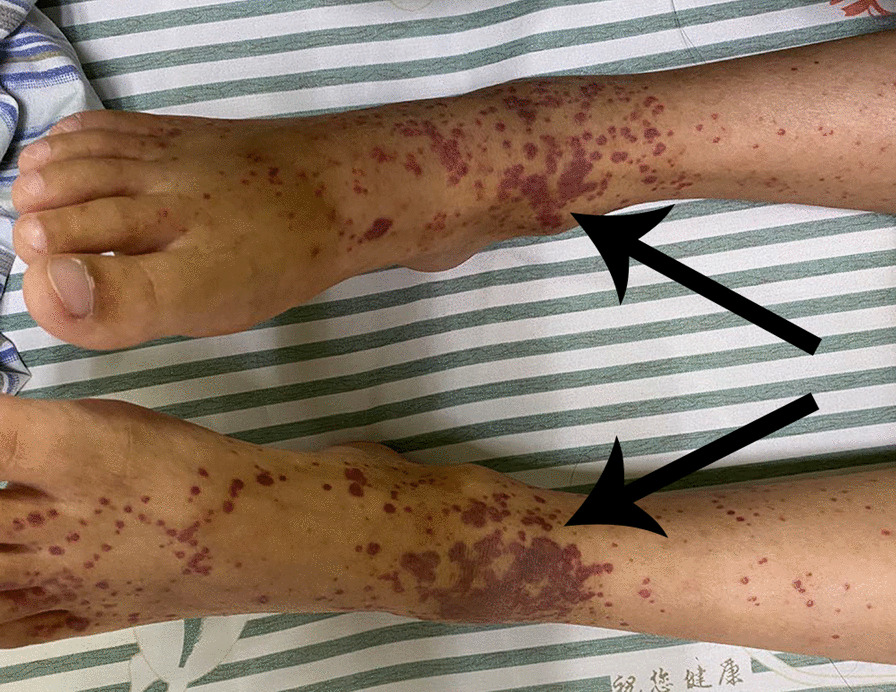
Typical palpable purpura on the lower limbs

**Fig. 2:**
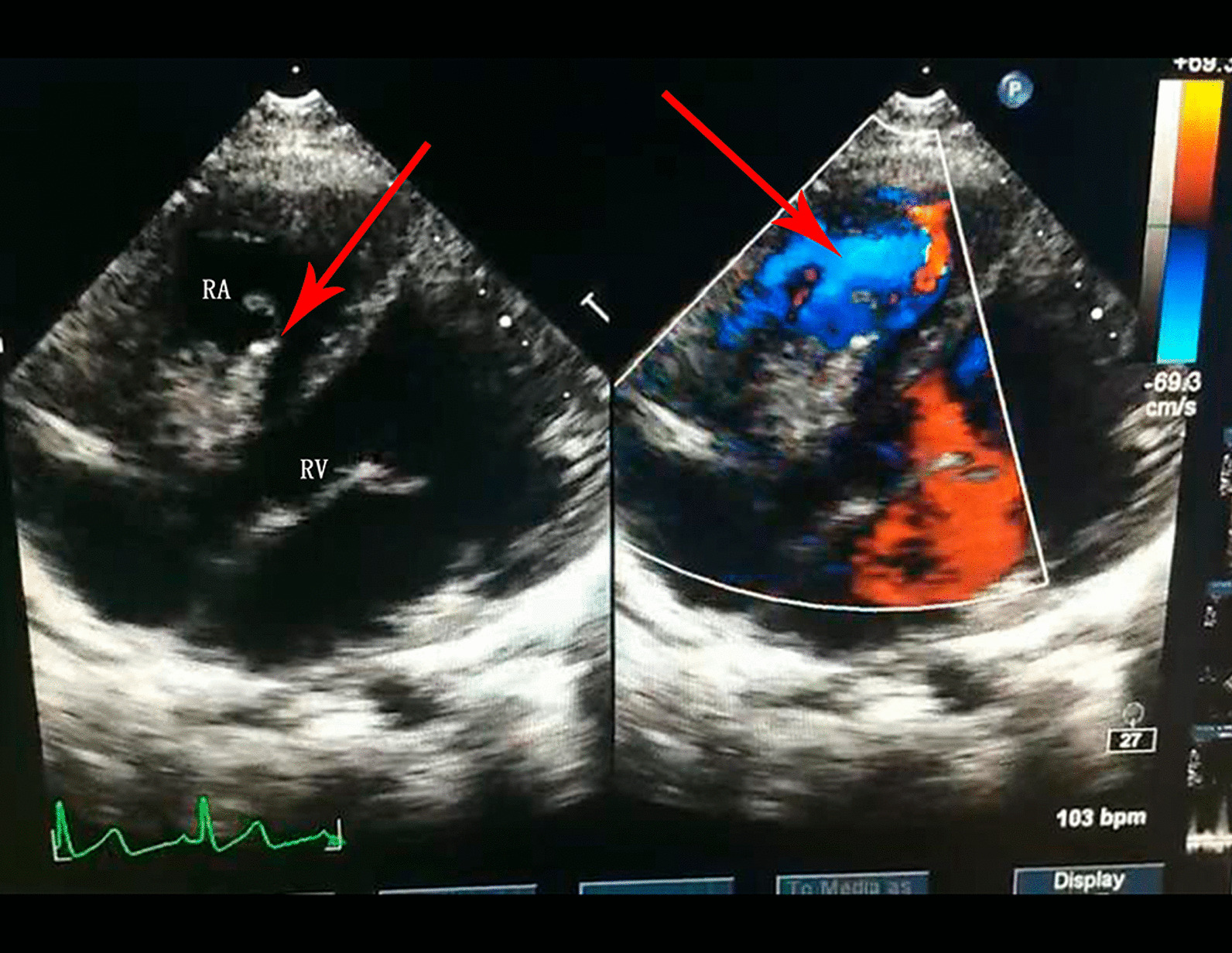
TTE showed signs of ventricular septal defect associated with aneurysm of the membranous septum, patent foramen ovale, mild pulmonary hypertension and severe tricuspid regurgitation with multiple irregular vegetations (larger about 24.5 mm × 8.3 mm and 22.7 mm × 0.69 mm) on the right ventricular side of the ventricular septal defect and on the tricuspid valve leaflets

A 10-year-old girl, with uncorrected congenital heart defects, was initially presented to our pediatric outpatient clinic because of the diffuse purple petechial rash on her lower extremities. The first clinical evaluation showed a positive urinalysis for red blood cell (RBC) with 1 (normal range 0–8) but a negative for protein, she was diagnosed with Henoch-Schonlein purpura and received an oral cetirizine and prednisolone. Two months later, the petechial rash disappeared with negative urinalysis for RBC and protein. Prior to this admission, the girl had two fevers with pharyngeal hyperemia in 7 months. She received an oral cefprozil but did not follow the doctor's prescription for a transthoracic echocardiography (TTE). It was not until 3 days prior to admission that the girl was again advised to do the TTE due to a recurrence of the petechial rash (Fig. 1). The TTE (Fig. 2) showed signs of ventricular septal defect associated with aneurysm of the membranous septum, patent foramen ovale (PFO), mild pulmonary hypertension alongside severe tricuspid regurgitation with multiple irregular vegetations (larger about 24.5 mm × 8.3 mm and 22.7 mm × 0.69 mm) on the right ventricular side of the ventricular septal defect and on the tricuspid valve leaflets. During hospitalization, the three blood cultures on days 1, 6, and 13 of admission grew *Abiotrophia defectiva*. Laboratory results are in Table 1. Initially, the girl was treated with intravenous meropenem. Later, vancomycin was added for a relapse of fever. Repeated TTE revealed similar results with a slight enlargement of the vegetations with 27.7 mm × 7.7 mm and 24.1 mm × 4.3 mm. Doppler ultrasonography showed hepatosplenomegaly.

**Table 1:** Laboratory data on inpatient test

	Patient value	Normal range
WBC (× 10^9^/L)	9.54	3.5–9.5
Hemoglobin (g/L)	82	115–150
Hematocrit (%)	26.9	35–45
Platelet (× 10^9^/L)	131	125–350
Urea (mmol/L)	15.20	2.50–6.10
Creatinine (umol/L)	6.90	53–106
Albumin (g/L)	34.7	35–50
ASO (IU/ml)	34	0–200
ESR (mm/h)	64	0–20
C3 (g/L)	0.17	0.87–1.41
c-ANCA	Negative	Negative
p-ANCA	Negative	Negative
ANA	Negative	Negative
*Urinalysis*		
RBC (/ul)	1685	0–8
Dysmorphic RBC (%)	11	
WBC (/ul)	19	0–14
Protein (g/L)	2 +	Negative

*WBC* white blood count; *ASO* antistreptolysin O test; *ESR* erythrocyte sedimentation rate; *C3* complement component 3; *ANCA* anti-neutrophilic cytoplasmic antibody; *ANA* anti-neutrophilic antibody; *RBC* red blood cell

**Fig. 3:**
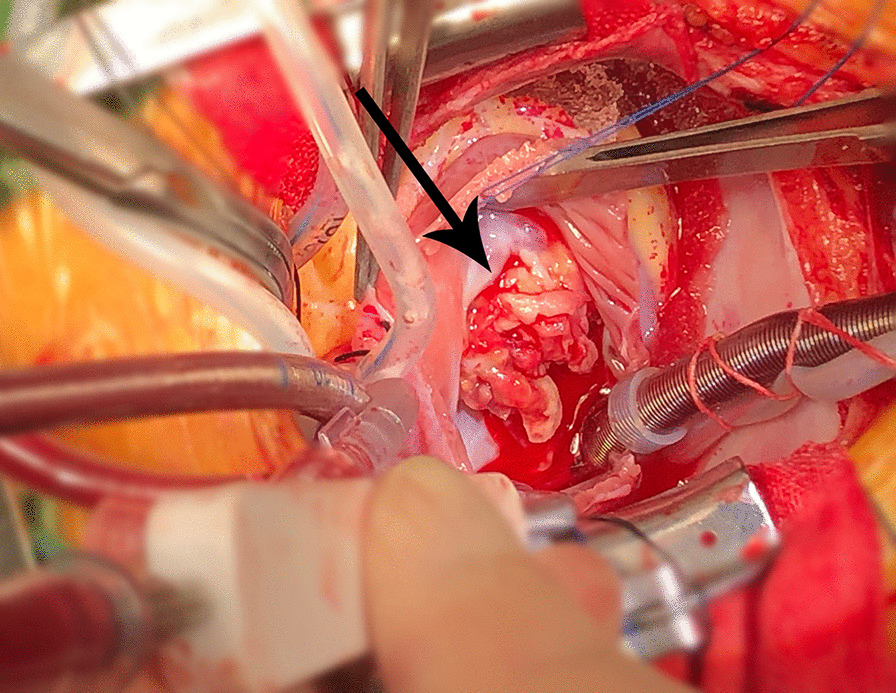
Open cardiac surgery image of the large vegetations on tricuspid valve

**Fig. 4:**
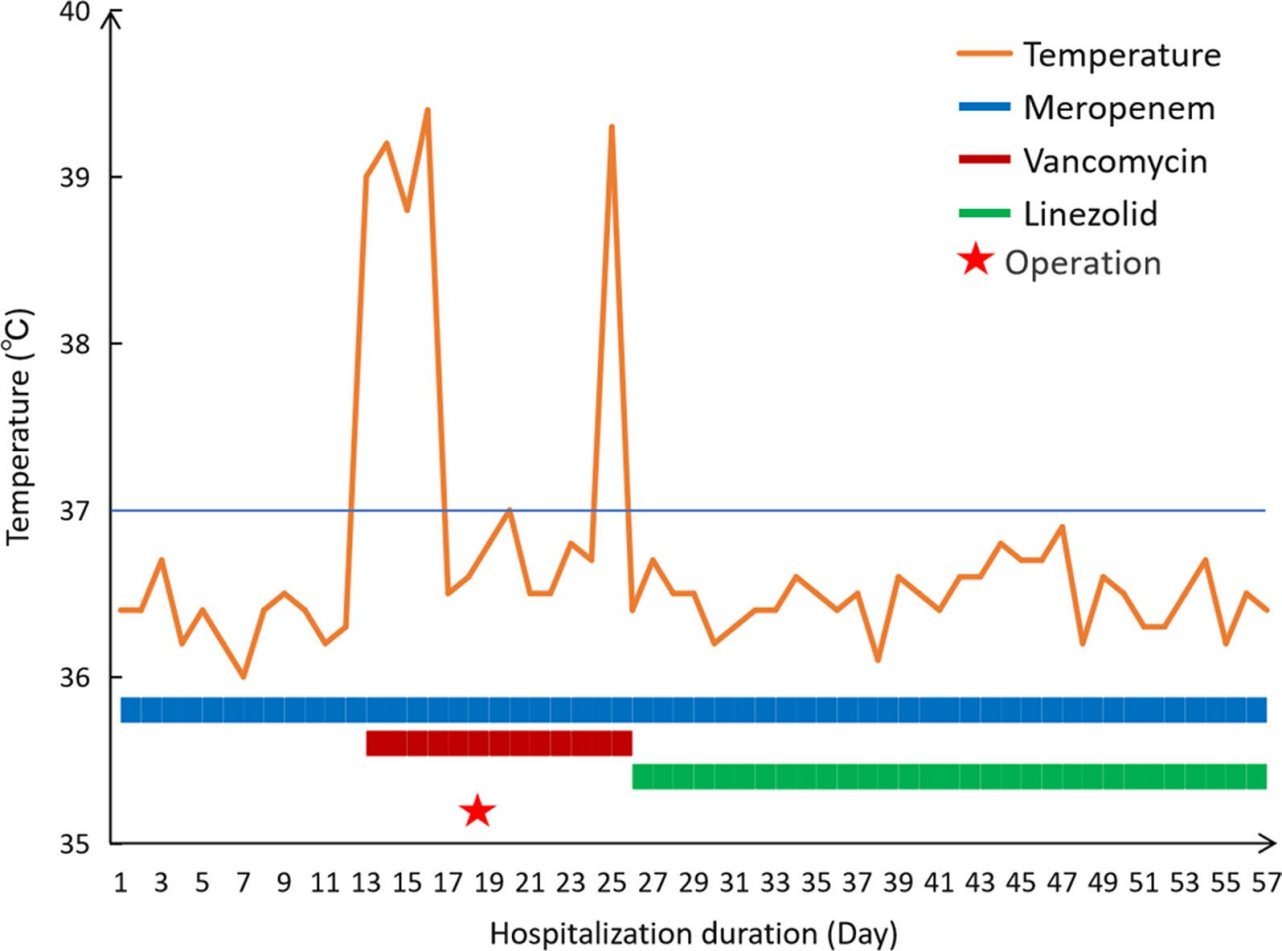
The process diagram of hospitalisation

On the 18th day, transthoracic cardiac surgery was performed with cardiopulmonary bypass. Intraoperatively, PFO and perimembranous ventricular septum defect (3 mm × 5 mm) were observed with the floppy and yellowish vegetations on the all 3 leaflets and chordae tendineae of the tricuspid valve (Fig. 3) and right ventricular side of VSD. Radical debridement of the vegetations and removal of the infected and necrotic leaflets and chordae tendineae tissue of tricuspid valve were performed. The VSD was closed with bovine pericardial patch and 4/0 polypropylene sutures, followed by the replacement of the tricuspid valve with bioprosthesis (27 mm) and closing of oval foramen. The aortic cross-clamp and cardiopulmonary bypass were 68 and 110 min, respectively. The vegetation culture showed no bacterial growth, and postoperative pathological examination of intraoperatively collected tissue samples revealed partial regional necrosis of the valve with abscess cavity and vegetation, and necrotic tissue, inflammatory exudate, and granulation tissue were observed in the vegetations. Blood cultures were repeated on postoperative days 2, 7 and 8, respectively, and none showed bacterial growth. In addition, 2 days before her discharge, a blood culture was performed again, still, no bacterial growth. The patient recovered well with an intensive care unit stay of 83 h and was discharged on postoperative day 39. The antibiotic regimen is shown in Fig. 4. Post-operatively, the patient received oral warfarin to maintain an International Normalized Ratio (INR) of approximately 2.0. Five months after the surgery, the patient was in satisfactory condition without any signs of endocarditis or valve insufficiency and her purpuric rash disappeared. Besides, the result of follow-up at one year showed that she was in good condition as well.

## Discussion

In practice, the diagnosis of HSP is usually based on the typical clinical presentation. It is essential to confirm leukocytoclastic vasculitis or proliferative glomerulonephritis with predominant deposition of IgA on histology, especially for patients with an unclear diagnosis or severe renal damage [1]. Currently, the European SHARE initiative recommends that skin biopsy is not required for typical palpable purpura on the lower limbs and buttocks [3]. HSP in children is usually self-limited, with 1/3 cases of recurrent purpura associated with renal involvement [1]. In this paper, we present a quite rare pediatric case of recurrent HSP combined with IE, and only a few adult cases of IE-associated HSP have been reported in the English literature [4]. Unfortunately, it is difficult to determine when the IE developed without a complete cardiac evaluation during the one-year course of the HSP for this girl. At least, the recurrent purpura may have something to do with IE, as the disappearance of purpura following the cure of IE could confirm this possibility, although the underlying mechanism is unknown.

IE is infrequent in children with an incidence of 0.3 to 3.3 per 100, 000 children and adolescents [5]. In the past 2 decades, congenital heart disease (CHD) has replaced rheumatic heart disease as the major predisposition for IE in children [6]. *Abiotrophia defectiva* is a Gram-positive streptococcus that colonizes in the oral cavity, gastrointestinal tract and urogenital tract of healthy individuals. It rarely causes IE that predominantly occurs in adults with structural heart disease, immunosuppression, prosthetic valves and invasive operations (such as dental procedures) [7, 8]. Atypical clinical presentation and fastidious nutritional make delays in the diagnosis and treatment of IE due to *Abiotrophia defective* [9, 10]. In addition, *Abiotrophia defectiva* is also a rare invasive organism predisposed to valve destruction, heart failure, systemic embolization and death as compared to other streptococci [10, 11]. Approximately 50% of patients with IE due to *Abiotrophia defectiva* require surgical intervention despite treatment with sensitive antibiotics [10].

IE caused by *Abiotrophia defectiva* is quite rare in pediatric cases, and it seems to be more devastating when compared to in adults. Since the definition of *Abiotrophia defectiva* was revised in 1995, including this case, only a total of 10 patients have been reported in the English medical literature [12]. The analysis of risk factors showed 4 cases (40%) with underlying CHD and 4 cases (40%) with a history of dental procedure. Right-sided valves were involved in 5 cases (50%), 4 of which had underlying CHD. Most patients present with a subacute course of atypical symptoms. In addition, nine of the ten (90%) cases developed complications, 7 (70%) of whom underwent surgery, while 4 of the 7 surgical patients underwent valve replacement due to severe valve destruction.

Valve replacement in children is always a challenge for cardiac surgeons because of the initial valve implant, small patient size, anatomy and growth potential. Without question, valvular repair is the goal of intervention because restoration of valvular anatomy and physiology using native tissue allows for growth and a potentially better long-term outcome [13].

Due to the high morbidity of complications associated with tricuspid valve replacement, it is rarely required to replace the tricuspid valve, particularly in children. In this case, the tricuspid valve structure suffered irreparable destruction, so valve replacement became inevitable. A bioprosthetic tricuspid valve replacement was used as an alternative for this girl.

## Conclusion

The coexistence of HSP and IE is possible, although the underlying pathogenesis remains unclear. *Abiotrophia defectiva* is a rare but devastating organism in children with IE and it is necessary to perform a comprehensive cardiac and pathogenic evaluation as early as possible in children with atypical symptoms, especially those with high-risk factors such as CHD. Notably, bioprosthetic valve replacement is considered feasible for irreparable tricuspid valve in children.

## Data Availability

Please contact author for data requests.

## Abbreviations

HSP: Henoch-Schonlein purpura
IE: Infective endocarditis
VSD: Ventricular septal defect
RBC: Red blood cell
TTE: Transthoracic echocardiography
WBC: White blood count
ASO: Antistreptolysin O test
ESR: Erythrocyte sedimentation rate
C3: Complement component 3
ANCA: Anti-neutrophilic cytoplasmic antibody
ANA: Anti-neutrophilic antibody
CHD: Congenital heart disease

## References

[1] Trnka P (2013). Henoch-Schonlein purpura in children. J Paediatr Child Health.

[2] Vincent LL, Otto CM (2018). Infective endocarditis: update on epidemiology, outcomes, and management. Curr Cardiol Rep.

[3] Ozen S, Marks SD, Brogan P, Groot N, de Graeff N, Avcin T (2019). European consensus-based recommendations for diagnosis and treatment of immunoglobulin A vasculitis-the SHARE initiative. Rheumatology.

[4] Ha SE, Ban TH, Jung SM, Bae KN, Chung BH, Park CW (2015). Henoch-Schonlein purpura secondary to infective endocarditis in a patient with pulmonary valve stenosis and a ventricular septal defect. KOREAN J INTERN MED.

[5] Jortveit J, Klcovansky J, Eskedal L, Birkeland S, Døhlen G, Holmstrøm H (2018). Endocarditis in children and adolescents with congenital heart defects: a Norwegian nationwide register-based cohort study. ARCH DIS CHILD.

[6] Baltimore RS, Gewitz M, Baddour LM, Beerman LB, Jackson MA, Lockhart PB (2015). Infective endocarditis in childhood: 2015 update: a scientific statement from the American Heart Association. Circulation.

[7] Mosca AM, Mane F, Marques PC, Medeiros P (2021). Infective endocarditis by a rare and fastidious agent: *Abiotrophia defectiva*. BMJ Case Rep.

[8] Ramos JN, Dos SL, Vidal LM, Pereira PM, Salgado AA, Fortes CQ (2014). A case report and literature overview: *Abiotrophia defectiva* aortic valve endocarditis in developing countries. Infection.

[9] Bozkurt I, Coksevim M, Cerik IB, Gulel O, Tanyel E, Leblebicioglu H (2017). Infective endocarditis with atypical clinical feature and relapse by *Abiotrophia defectiva*. J Saudi Heart Assoc.

[10] Park S, Ann HW, Ahn JY, Ku NS, Han SH, Hong GR (2016). A Case of infective endocarditis caused by *Abiotrophia defectiva* in Korea. Infect Chemother.

[11] Pinkney JA, Nagassar RP, Roye-Green KJ, Ferguson T (2014). *Abiotrophia defectiva* endocarditis. BMJ Case Rep.

[12] Song SH, Ahn B, Choi EH, Lee SP, Cho EY, Bae EJ (2020). *Abiotrophia defectiva* as a cause of infective endocarditis with embolic complications in children. Infection.

[13] Henaine R, Roubertie F, Vergnat M, Ninet J (2012). Valve replacement in children: a challenge for a whole life. Arch Cardiovasc Dis.

